# Immune dampening effects of the vagus nerve in a model for food allergy

**DOI:** 10.1186/2045-7022-3-S3-P11

**Published:** 2013-07-25

**Authors:** A De Vries, PJ Gomez Pinilla, R De Keyser, G Matteoli, M Di Giovangiulio, GE Boeckxstaens

**Affiliations:** 1 TARGID, KU Leuven, Leuven, Belgium

## Background

In our model of mild food allergy, allergen encounter typically leads to mast cell degranulation, influx of Th2 immune cells together with increased levels of antigen specific antibodies. This leads to bowel function changes resulting in diarrhoea. Vagal nerve stimulation has immune-suppressive effects on innate immune responses. Here we tested whether vagal tone modulates adaptive immune responses in food allergy by applying vagal nerve stimulation (VNS) or vagotomy.

## Methods

We sensitised balb/c mice to ovalbumin (OVA) adsorbed to aluminium-hydroxide (day 0 and 14) followed by intra-gastric gavages with OVA, from day 28 onwards. The vagal nerve was stimulated at the cervical level (5V, 5Hz, 1ms, for 5mins) prior to each gavage, controls received sham surgery. The vagus nerve was cut (VGX) sub-diaphragmatically at least one week prior to OVA gavage. We determined antibody titres (OVA specific IgG1, IgG2a and IgE), mast cell proteases (mouse mast cell protease 1, MMCP-1) released into the circulation and identified immune cells in the mesenteric lymph nodes and lamina propria. Diarrhoea was scored for 1hr by analysing pellets.

## Results

VNS resulted in a reduction of serum mMCP-1 (1hr after the last OVA gavage, from 4038 ± 907 to 1093 ± 436 ng/mL (n=8, p=0.01) ) and IgG2a (relative serum dilutions, 2-way ANOVA F(7,84)=5.61 p<0.0001). Serum OVA IgG1 and IgE were unaffected. In addition, VNS increased the percentage of Tregs in the mesenteric lymph nodes compared to sham controls (from 10.2 ± 0.3 to 12.3 ± 0.2% CD25+/foxp3+ of CD4 cells, p< 0.0001), whereas Th1, Th17 T cells, Th2 T cells and the total number of T-cells remained unaffected. In contrast to VNS, VGX resulted in increased levels of serum mMCP-1 (1120 ± 136 vs 2841 ± 964 ng/mL (n=6/7, p<0.05) coinciding with increased diarrhoea scores, but no changes were detected in Tregs, Th17 or Th2 cells in the lamina propria, mesenteric lymph nodes or Peyer’s patches.

## Conclusion

Our results suggest that VNS improves food allergy by increasing the numbers of Tregs and reducing mast cell degranulation, whereas reduced vagal tone following VGX increases disease activity. These data suggest that the vagus nerve not only modulates the innate immune system, but also the adaptive immune system. Further insight in the mechanism of action may ultimately reveal novel drug targets to improve treatment of patients with food allergy.

## Disclosure of interest

None declared.

